# The Safety of CT-Guided Percutaneous Lung Biopsy in Elderly Patients With Solitary Pulmonary Nodules

**DOI:** 10.7759/cureus.44105

**Published:** 2023-08-25

**Authors:** Haibo Yang, Xiaofang Gao

**Affiliations:** 1 Department of Respiratory Medicine, Chinese People's Liberation Army 92493 Military Hospital, Huludao City, CHN; 2 Department of Lung Cancer, Beijing Arion Cancer Center, Beijing, CHN

**Keywords:** geriatric medicine, pneumothorax, pulmonary hemorrhage, cancer, solitary pulmonary nodules, the elderly, percutaneous lung biopsy

## Abstract

Objective: Computed tomography (CT)-guided percutaneous lung biopsy is an effective diagnostic procedure for patients with solitary pulmonary nodules (SPN). The aim of this study is to evaluate the safety of this procedure for elderly patients with SPN.

Methods: A total of 125 patients with SPN who received a CT-guided percutaneous lung biopsy were retrospectively analyzed. Patients were divided into elderly (age 65 and above) and non-elderly groups. The patients' characteristics and procedure-related complications were compared between the two groups.

Results: The elderly and non-elderly groups included 74 and 51 patients, respectively. The success rate of a CT-guided percutaneous lung biopsy was 100%. The diagnosis rate of lung cancer in the elderly group was significantly higher than that in the non-elderly group (83.78% vs. 64.70%, p = 0.014). The incidence of pulmonary hemorrhage after lung biopsy in the elderly group (44, 59.45%) was significantly higher than that in the non-elderly group (21, 41.17%, p = 0.044), and moderate hemorrhage was the main contributor. The incidence rate of pneumothorax in the elderly group numerically increased, but the difference did not reach statistical significance.

Conclusion: Computed tomography-guided percutaneous lung biopsy was an efficient procedure for diagnosing SPN in elderly patients. Although complication rates were relatively higher in elderly patients, the safety of this procedure was acceptable.

## Introduction

A solitary pulmonary nodule (SPN) is defined as a relatively well-defined round or oval pulmonary parenchymal lesion equal to or smaller than 30 mm in diameter [1]. With the coming of an aging society, the number of elderly patients with SPN is increasing by the day. The tolerance of elderly patients to thoracic operations is unsatisfactory. Therefore, a preoperative, definite diagnosis to avoid unnecessary surgery is important for elderly patients with SPN [2].

Computed tomography (CT)-guided percutaneous lung biopsy is an effective diagnostic procedure for SPN, but its safety in elderly patients needs further understanding and clarification [3]. The purpose of our study was to investigate the clinical safety of CT-guided percutaneous lung biopsy of SPN in elderly patients.

## Materials and methods

Study population

We retrospectively collected electronic medical records of 125 patients diagnosed with SPN who underwent CT-guided percutaneous lung biopsy at the Chinese People's Liberation Army 92493 Military Hospital, Huludao City, and Beijing Arion Cancer Center, Beijing, China, from July 2022 to July 2023. The protocol of this retrospective study was approved by the institutional review board, with a waiver of informed consent obtained. Patients were divided into elderly (age 65 and above) and non-elderly groups. Patients’ data about age, gender, smoking, previous chronic obstructive pulmonary disease (COPD), and pulmonary nodule size were collected.

Biopsy procedure

The procedure of a CT-guided percutaneous lung biopsy was performed by experienced interventional radiologists. Patients received an enhanced CT scan before the procedure to avoid injury to larger vessels. An 18-gauge coaxial automated cutting needle and a localization needle (Bard Max-Core biopsy needle; C.R. Bard, Inc., New Providence, NJ, USA) were used for the biopsy. The patients received a repeated CT scan after the biopsy to assess procedure-related complications. The occurrence of pulmonary hemorrhage and pneumothorax after lung biopsy was confirmed by medical records and postoperative imaging. Postoperative imaging showing stripe hemorrhage along the needle tract was identified as mild hemorrhage, and flake hemorrhage was identified as moderate hemorrhage [4]. A large pneumothorax was defined on erect chest radiographs as an air space that was more than 2 cm from the pleural surface to the lung edge at the level of the hilum; otherwise, it was a small pneumothorax [5].

Statistical analysis

The patients' characteristics and procedure-related complications were compared between the elderly and non-elderly groups. Categorical variables were shown as numbers (percentages). Continuous variables were expressed as means with standard deviations. To compare the two patient groups, t-tests, and chi-square tests were used for normally distributed and nominal data between groups. Significance was defined as a p-value less than 0.05, and all tests were two-sided. Data were analyzed using the IBM SPSS software (Version 26.0; IBM Corp., Armonk, NY, USA).

## Results

A total of 125 patients, including elderly (n=74) and non-elderly (n=51) patients, were retrospectively analyzed. There was no significant difference in gender, smoking, or nodule size between the elderly and non-elderly groups. The patients with COPD in the elderly group significantly outnumbered the patients in the non-elderly group (p = 0.038). The results are shown in Table 1.

**Table 1:** Characteristics of the patients in the elderly and non-elderly group COPD: chronic obstructive pulmonary disease

	Male, n(%)	Age, years	Smoking, n(%)	COPD, n(%)	Nodule size, mm
Elderly, n=74	51 (69.91)	71.05±8.62	31 (41.89)	13 (17.56)	18.72±4.59
Non-elderly, n=51	32 (62.74)	51.42±8.59	19 (37.25)	2 (3.92)	17.83±5.07
p-value	0.472	<0.01	0.603	0.021	0.468

The success rate of a CT-guided percutaneous lung biopsy was 100%. In the elderly group, 62 patients were diagnosed with lung cancer after a lung biopsy. Adenocarcinoma (44, 70.96%), squamous carcinoma (11, 17.74%), and small lung cell cancer (5, 8.06%) were the top three pathological types. In the non-elderly group, 33 patients were diagnosed with lung cancer. The numbers of patients with adenocarcinoma, squamous carcinoma, and small lung cell cancer were 24 (72.72%), four (12.12%), and two (6.06%), respectively (Table 2).

**Table 2:** Comparison of pathological types between elderly and non-elderly patients diagnosed with lung cancer

	Adenocarcinoma, n(%)	Squamous carcinoma, n(%)	Small lung cell cancer, n(%)
Elderly, n=62	44 (70.96)	11 (17.74)	5 (8.06)
Non-elderly, n=33	24 (72.72)	4 (12.12)	2 (6.06)
p-value	0.856	0.474	0.721

The diagnosis rate of lung cancer in the elderly group was significantly higher than that in the non-elderly group (83.78% vs. 64.70%, p = 0.014).

The incidence of pulmonary hemorrhage after lung biopsy in the elderly group (44, 59.45%) was significantly higher than that in the non-elderly group (21, 41.17%, P = 0.044). Subgroup analysis showed the significant difference was attributed to moderate hemorrhage rather than mild hemorrhage (Table 2). The incidence rate of pneumothorax in the elderly group (31, 41.89%) was numerically higher than that in the non-elderly group (13, 25.49%), but the difference did not reach statistical significance (p = 0.059). Subgroup analysis of large and small pneumothorax came to the same conclusion (Table 3).

**Table 3:** Comparison of complications between the elderly and non-elderly groups

	Pulmonary hemorrhage		Pneumothorax	
	Mild, n(%)	Moderate, n(%)	Small, n(%)	Large, n(%)
Elderly, n=74	23 (31.08)	21 (28.37)	26 (35.13)	5 (6.75)
Non-elderly, n=51	17 (33.33)	4 (7.84)	11 (21.56)	2 (3.92)
p-value	0.790	0.020	0.102	0.498

## Discussion

Solitary pulmonary nodules tended to be found more often in the elderly population with the development of imaging technology. Previous studies demonstrated that older patients with SPN were at higher risk of lung cancer [6]. Our study also indicated that the probability of malignancy in solitary pulmonary nodules was significantly increased in elderly patients. In light of this, using an effective method to distinguish the benign or malignant SPN was an important factor in improving the outcome of those patients [7].

Pathological examination was the gold standard for the diagnosis of SPN. A mandatory prerequisite for pathological diagnosis was to obtain tissue specimens from the lesion [8]. Computed tomography-guided lung biopsies were first reported by Haaga and Alfidi in 1976. This procedure has been constantly perfected with the development of percutaneous puncture and imaging technologies over the years [9]. However, the safety of this procedure for elderly patients still needs to be illustrated.

Pneumothorax and pulmonary hemorrhage were the main complications of a CT-guided percutaneous lung biopsy. Meta-analysis showed that the pooled complication rates of core needle lung biopsy were 38.8% (34.3%-43.5%) [10]. The risk of complications was related to the location and size of the SPN, the thickness of the puncture needle, the experience and skills of the operator, and the concomitant diseases of the patients [11].

Our study showed the occurrence rate of pulmonary hemorrhage in elderly patients significantly increased, and moderate hemorrhage was the major driving force. Gas exchange was the main function of the lung, and the blood supply was very rich in this organ. It was almost impossible to avoid pulmonary vascular injury during a percutaneous lung biopsy. In elderly patients, the increased vascular fragility and decreased vasoconstriction after puncture injury might result in an increased incidence of moderate hemorrhage [12]. In addition to this, the increasing use of aspirin in elderly patients might be another reason [13].

As lung compliance gradually reduces with age, emphysema and other lung problems in elderly patients also increase [14]. Our study showed that the number of patients with COPD in the elderly group significantly increased. Previous studies indicated that the risk of pneumothorax after percutaneous lung biopsy significantly increased in patients with COPD, pulmonary bulla, and pulmonary fibrosis [15]. In our study, the incidence rate of pneumothorax in the elderly group was numerically higher than that in the non-elderly group, but the difference did not reach statistical significance. The higher incidence of pneumothorax might be associated with the higher prevalence of COPD in elderly groups.

Our study has several limitations that should be taken into account. For example, complete data collection is impaired by a retrospective study design, which in turn leads to the impossibility of exploring the risk factors associated with the complications. The present study is representative of a small group of elderly patients, and further large-scale clinical trials with prospective designs should be carried out to confirm the findings.

## Conclusions

A CT-guided percutaneous lung biopsy was an efficient procedure for diagnosing SPN in elderly patients. Although complication rates were relatively higher in elderly patients, the safety of CT-guided percutaneous lung biopsy was acceptable, and most of the complications were mild adverse events. There should be no hesitation in performing a CT-guided percutaneous lung biopsy in elderly patients with SPN because of the irreplaceable role of this procedure in pathological diagnosis.
